# Influence of different degrees of head elevation on respiratory mechanics in mechanically ventilated patients

**DOI:** 10.5935/0103-507X.20150059

**Published:** 2015

**Authors:** Bruno Prata Martinez, Thaís Improta Marques, Daniel Reis Santos, Vanessa Silva Salgado, Balbino Rivail Nepomuceno Júnior, Giovani Assunção de Azevedo Alves, Mansueto Gomes Neto, Luiz Alberto Forgiarini Junior

**Affiliations:** 1 Hospital Aliança - Salvador (BA), Brazil.; 2 Universidade do Estado da Bahia - Salvador (BA), Brazil.; 3 Hospital Santo Antônio, Obras Sociais Irmã Dulce - Salvador (BA), Brazil.; 4 Universidade Federal da Bahia - Salvador (BA), Brazil.; 5 Postgraduate Program, Universidade Cidade de São Paulo - São Paulo (SP), Brazil.; 6 Centro Universitário Metodista - IPA - Porto Alegre (RS), Brazil.

**Keywords:** Respiratory mechanics, Inpatients, Patient positioning, Intensive care units

## Abstract

**Objective:**

The positioning of a patient in bed may directly affect their respiratory mechanics. The objective of this study was to evaluate the respiratory mechanics of mechanically ventilated patients positioned with different head angles hospitalized in an intensive care unit.

**Methods:**

This was a prospective physiological study in which static and dynamic compliance, resistive airway pressure, and peripheral oxygen saturation were measured with the head at four different positions (0° = P1, 30° = P2, 45° = P3, and 60° = P4). Repeated-measures analysis of variance (ANOVA) with a Bonferroni post-test and Friedman analysis were used to compare the values obtained at the different positions.

**Results:**

A comparison of the 35 evaluated patients revealed that the resistive airway pressure values in the 0° position were higher than those obtained when patients were positioned at greater angles. The elastic pressure analysis revealed that the 60° position produced the highest value relative to the other positions. Regarding static compliance, a reduction in values was observed from the 0° position to the 60° position. The dynamic compliance analysis revealed that the 30° angle produced the greatest value compared to the other positions. The peripheral oxygen saturation showed little variation, with the highest value obtained at the 0° position.

**Conclusion:**

The highest dynamic compliance value was observed at the 30° position, and the highest oxygenation value was observed at the 0° position.

## Abstract (pt)

**Objetivo:**

O posicionamento do paciente no leito pode afetar diretamente a mecânica respiratória. Este estudo teve como objetivo avaliar a mecânica respiratória em diferentes angulações da cabeceira em pacientes internados na unidade de terapia intensiva sob ventilação mecânica.

**Métodos:**

Trata-se de um estudo prospectivo fisiológico, no qual foram mensuradas a complacência estática e dinâmica; a pressão resistiva das vias aéreas e saturação periférica de oxigênio nas diferentes posições adotadas (0° = P1, 30° = P2, 45° = P3 e 60° = P4). Para comparação dos valores obtidos nas diferentes posições, foi utilizada a Análise de Variância de medidas repetidas (ANOVA) com pós-teste de Bonferroni e análise de Friedman.

**Resultados:**

Quando comparamos os 35 pacientes avaliados, os valores da pressão resistiva das vias aéreas na posição a 0° foram superiores em relação às angulações mais elevadas. Já na análise da pressão elástica, a posição a 60° apresentou o maior valor em relação às outras posições. Em a relação à complacência estática, houve redução dos valores da posição 0° para a posição 60°. Quando analisada a complacência dinâmica, observou-se que a angulação de 30° apresentou o maior valor, quando comparada às demais posições. A saturação periférica de oxigênio apresentou pequena variação, sendo o maior valor obtido na posição 0°.

**Conclusão:**

A complacência dinâmica apresentou maior valor na posição a 30° em relação às outras angulações, sendo que a posição de maior oxigenação foi a 0°.

## INTRODUCTION

The positioning of a patient in bed can directly affect respiratory function in mechanically ventilated (MV) patients.^(1,2)^ The posture imposed on MV patients may facilitate diaphragmatic performance, but it may also increase the mechanical load against the respiratory system airflow.^(3,4)^

The current recommendation is that the head of MV patients should be maintained between 30° and 45° because of the high risk of bronchoaspiration and because this position can reduce the risk of mechanical ventilation-associated pneumonia.^(5,6)^ In addition to promoting a reduction in the risk of developing pneumonia, some postural positions can increase the possibility of more homogeneous alveolar ventilation and possibly reduce the risk of lung injury caused by mechanical ventilation similar to that in patients undergoing ventilation in the prone position.^(7)^

Although the effects of positioning the head at 30° and 45° on the reduction of mechanical ventilation-associated pneumonia are known, no studies have evaluated the difference in mean values obtained for mechanical ventilation at different head angles in this population. Therefore, the objective of this study was to evaluate the respiratory mechanics of MV patients admitted to the intensive care unit (ICU) who were positioned with different head angles (0°, 30°, 45°, and 60°).

## METHODS

This was a prospective physiological study conducted in the ICU of the *Hospital Santo Antônio, Obras Sociais Irmã Dulce*, in the city of Salvador (BA), between October 2009 and January 2010. The study included adult patients of both genders who were over 18 years of age, in the ICU for more than 24 hours, undergoing invasive MV, sedated, not interacting with the mechanical ventilator, which was visualized by graphical analysis, and hemodynamically stable, characterized by the absence or low doses of vasoactive or inotropic drugs. Patients with recent fractures (chest wall, spine, and hip) and those with a clinical diagnosis of pulmonary fibrosis or acute respiratory distress syndrome were excluded. Patients who showed changes in mean arterial pressure greater than 20% relative the baseline value, a systolic blood pressure < 90mmHg in invasive blood pressure measurements, and peripheral oxygen saturation < 90% during mechanical measurements were also excluded. The present study was approved by the Research Ethics Committee of the *Hospital Santo Antônio* (protocol number 46/09). Individuals who were responsible for the patients were informed about the study and signed an informed consent form authorizing participation.

The measured values of respiratory mechanics were obtained from a TBIRD VELA mechanical ventilator (Viasys Respiratory Care, United States) and included respiratory system static (Cst, rs) and dynamic (Cdyn, rs) compliance and resistive airway pressure. Hemodynamic data such as mean arterial pressure, systolic blood pressure, heart rate, and peripheral oxygen saturation were obtained from a multiparameter monitor (DIXTAL, Manaus, Brazil).

The patients included in the study were evaluated at four different positions (0° = P1, 30° = P2, 45° = P3, and 60° = P4), which were randomly allocated, and randomization of the positions was conducted in a point by point manner. For greater accuracy, a goniometer was used to verify the head angle adopted for each position.

Before the evaluation of respiratory mechanics, a single alveolar recruitment maneuver was performed for pulmonary homogenization, with patients in a pressure controlled ventilation mode with a 100% inspired oxygen fraction and an increased positive end expiratory pressure (PEEP) of 2cmH_2_O every minute until a value of 20cmH_2_O was reached. This condition was maintained for two minutes and followed by reduction of 2cmH_2_O per minute until the initial PEEP level was achieved.^(8)^ After 30 minutes, the patients were placed in a controlled volume ventilation mode for evaluation of respiratory mechanics with the following parameters: tidal volume of 6 - 8mL/kg in relation to the ideal weight, 40 L/min flow, square wave flow, a respiratory rate of 15 breaths per minute, and an inspiratory pause time of 0.5 seconds.^(9)^ These parameters were maintained for approximately two minutes in each position, and the peak and plateau pressure values and the mean PEEP were recorded. The screen was paused to record the peak and plateau pressures; the highest value was considered the peak, and the pressure value closest to the 0.5-second pause time and with a flow equal to zero was recorded as the plateau.

Static compliance was calculated by dividing the tidal volume by the respiratory system elastic pressure or driving pressure (plateau pressure subtracted from the mean PEEP value). For dynamic compliance, the tidal volume was divided by the peak pressure subtracted from the mean PEEP value. Resistive airway pressure was calculated as the difference between the peak and plateau pressures.

The data are described as means and standard deviations for variables with a normal distribution and as medians and interquartile ranges for data with a non-normal distribution. Data normality was measured using the Shapiro-Wilk test. The distribution was normal only for resistive pressure, and in this case, repeated-measures analysis of variance (ANOVA) with a Bonferroni post-test was used. For other variables (elastic pressure, Cst, rs; Cdyn, rs, and peripheral oxygen saturation) with non-normal distributions, the nonparametric Friedman test was used. The significance level was p < 0.05. All analyses were performed using the Statistical Package for Social Sciences (SPSS) version 14.0.

## RESULTS

**Figure 1:**
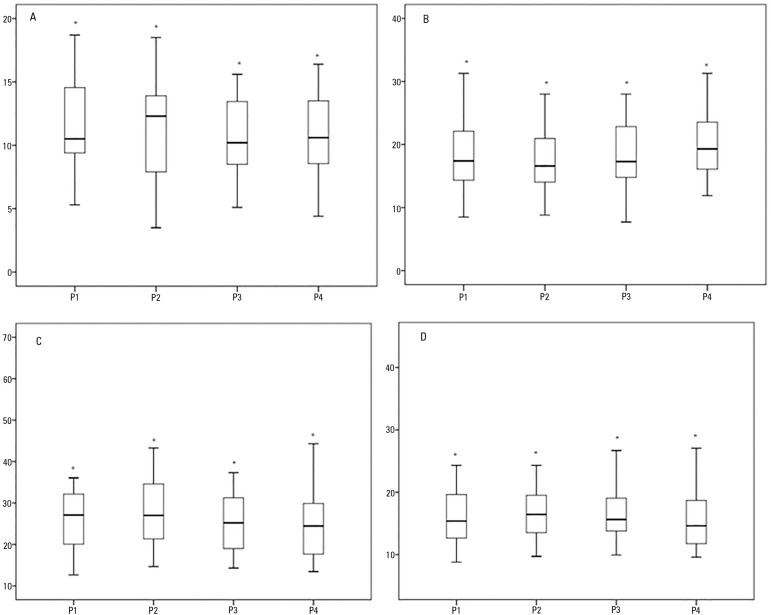
Analysis of resistive airway pressure (A), respiratory system elastic pressure (B), respiratory system static compliance (C), and respiratory system dynamic compliance (D) in the four positions (P1 = 0º, P2 = 30º, P3 = 45º and P4 = 60º). * p value < 0.05.

During the data collection period, 35 patients were included in the study, of whom 27 (77.7%) had a primary diagnosis of pneumonia and eight (22.3%) were undergoing a postoperative period after abdominal surgery. The mean age was 58.1 ± 15.6 years, and 66.6% of the patients were male. No complications, such as peripheral oxygen saturation below 90% or hemodynamic changes, were reported during the procedures. Table 1 and figure 1 show the static and dynamic compliance, resistive airway pressure, and alveolar distension pressure values.

**Table 1:** Evaluation of respiratory mechanics variables at different body positions

Variable	Position	Values
Resistive pressure (cmH_2_O)	P1	11.6 ± 3.5 (10.4 - 12.8)*
	P2	10.9 ± 3.8 (9.6 - 12.2)
	P3	10.8 ± 3.2 (9.6 - 11.9)
	P4	10.8 ± 3.5 (9.6 - 12.0)
Elastic pressure (cmH_2_O)	P1	17.4 (14.0 - 22.1)
	P2	16.6 (13.7 - 21.4)
	P3	17.3 (14.8 - 23.8)
	P4	19.3 (16.1 - 25.3)*
Cst, rs (mL/cmH_2_O)	P1	27.1 (19.5 - 32.3)
	P2	27.0 (20.7 - 34.8)
	P3	25.2 (18.4 - 31.5)
	P4	24.5 (17.4 - 29.9)*
Cdyn, rs (mL/cm H_2_O)	P1	15.4 (12.3 - 20.2)
	P2	16.4 (12.7 - 19.5)*
	P3	15.6 (13.7 - 19.1)
	P4	14.6 (11.8 - 18.7)
SpO_2_ (%)	P1	97.0 (96.0 - 98.0)
	P2	97.0 (96.0 - 98.0)
	P3	96.0 (96.0 - 97.0)
	P4	96.0 (96.0 - 97.0)

Cst, rs - static compliance; Cdyn, rs - dynamic compliance; SpO_2_ - pulse oximetry. Results are expressed as the mean ± standard deviation (95% confidence interval) or median (25%-75%).

* p < 0.001.

A comparison of resistive pressure values revealed that the 0º position values were higher than those recorded for greater angles (Table 1). The elastic pressure analysis revealed that the 60º position produced the highest value of all positions (p = 0.001).

Regarding the Cst, rs, a significant reduction in values was observed from the 0º position to the 60º position (p = 0.001). An analysis of the Cdyn, rs revealed that the 30º angle produced the highest value of all positions (p = 0.001). Peripheral oxygen saturation did not differ significantly when the 0º and 60 º positions were compared (p = 0.465).

## DISCUSSION

A change in the angle of the head affects the respiratory mechanics of MV patients. In this study, the largest resistive pressure value was found at the 0º position, and the largest value of elastic pressure was found at the 60º position. For the Cdyn, rs, the highest value occurred in the 30º position.

In an intervention study involving early mobilization of intubated abdominal surgery patients, Zafiropoulos et al.^(10)^ observed that high thoracic positions, such as sitting upright for 20 minutes, led to an improvement in transthoracic pressure, with consequent improvement in the Cst, rs. This gain enabled a reduction in the driving pressure required for the generation of a similar lung volume. This knowledge is crucial and must be employed in ventilatory lung protection strategies. Such differences may be relevant to clinical practice because variations in driving pressure, for example, may be associated with lower mortality in patients with and without acute respiratory distress syndrome, which has been demonstrated in recent meta-analyses.^(11,12)^

In the present study, the lowest driving pressure was observed at the 30º position, but the values were higher than 15cmH_2_O, which is not consistent with current recommendations for ventilatory strategy in MV patients.^(13)^ One possible explanation for this finding is that when the study was conducted, that recommendation did not exist, and patients were ventilated with tidal volumes of 6 to 8mL/kg because they had not been diagnosed with acute respiratory distress syndrome.

Although the literature reports improved respiratory system compliance in the sitting position compared to the dorsal and lateral decubitus positions,^(8)^ our study revealed a reduction of these values at greater angles, possibly due to the higher transthoracic pressure. However, it is not possible to state that this finding results from an increase in intra-abdominal pressure, as this variable was not evaluated; however, in all of our measurements, the legs were parallel to the ground to prevent further tilting with higher head positions.

To distinguish this possible chest wall change from a pulmonary change, it would be necessary to measure transpulmonary pressure, which would require the use of an esophageal balloon to estimate the pleural pressure value. Thus, the mechanical variations obtained at different positions could be related to variations in pleural pressure (thoracic) or alveolar pressure changes (pulmonary).^(9)^

Similarly, airway resistance was influenced by body position, with the highest value found with the head at 0º. This result was not expected because it had been thought that the lowest resistance would be found in the pulmonary areas of greatest respiratory system compliance. However, the opposite result was observed because greater resistance was found at the position of greatest compliance, which indicates that no inverse linear relationship exists between these two variables.^(14,15)^

In the evaluation of the Cdyn, rs, the highest value was observed at the 30º position, which may be explained by the close relationship between reduced resistive pressure and increased elastic pressure. A possible explanation for this finding is the reduction in resistance due to an increase in functional residual capacity (FRC), as well as a reduction in the intrathoracic blood volume.^(14,16)^ In addition to likely facilitating ventilation, this angle reduces the risk of mechanical ventilation-associated pneumonia.^(5,6)^

In addition to maintaining a body position for a prolonged time, the weight of the lung, when associated with an inflammatory process, facilitates the creation of dependent zones, with a decrease in compliance and an increase in resistance.^(17)^ This reduced compliance is associated with an increased risk of death.^(18)^ Positions facilitating a reduction in mechanical load that opposes the entry of air are therefore fundamental for greater stabilization of the air in the alveoli.^(19-21)^

The quasi-static method used to measure respiratory mechanics with occlusion at the end of inspiration was chosen due to the ease of bedside application in critically ill patients, but this method cannot differentiate between the chest wall and lung components.^(22)^ For this purpose, the use of invasive methods that can quantify pleural pressure is necessary.^(23)^

The increased peripheral saturation at the 0º position can be explained by the movement of blood to areas with a better ventilation/perfusion ratio that have proportionately greater vascularity at the posterior region of the lung, which remains dependent in the supine position at 0º.^(22)^ However, the 0.6% difference was not statistically significant.

In the case of obese patients, increased intra-abdominal pressure and general anesthesia can also cause changes in respiratory mechanics. An increase in intra-abdominal pressure increases chest wall elastance, reduces compliance, and promotes cranial displacement of the diaphragm. These factors may explain the higher esophageal pressure values in overweight/obese individuals. In obese patients, increased intra-abdominal pressure is the major determinant of lung volume reduction and premature closure of the small airways, especially when associated with anesthesia, which increases the reduction in functional capacity.^(24-27)^

In the present study, the alveolar recruitment maneuver was performed to homogenize the lung before applying the different head inclinations, thereby ensuring that the behavior of the variables of interest exhibited less bias due to possible gain or loss of alveolar unit recruitment between position changes.

This study had some limitations, such as the lack of non-invasive, intra-abdominal pressure measurements and the absence of pleural pressure measurements, which may also be a confounding variable in respiratory mechanics. In addition, mortality scores, cumulative fluid balance, use of vasopressor and inotropic drugs, use of renal replacement therapy, total mechanical ventilation time, hospital survival, and mean tidal volume were not measured. However, because this is the first study to evaluate different head angles with respect to respiratory mechanics, additional studies are needed to evaluate the effect of these variables. Another limitation was that the evaluated population had a very heterogeneous profile, which, although mostly consisting of patients with pneumonia, also included patients in the postoperative period after abdominal surgery. As stated previously, the driving pressure and tidal volume values were higher than the current recommendation, which is also a possible limitation of this study. Further studies evaluating the elastic components of the respiratory system that can affect these changes, such as intra-abdominal, chest wall, and pulmonary pressure and the ventilation versus infusion relationship, are needed.

## CONCLUSION

Head angle affected the respiratory mechanics of mechanically ventilated patients. The highest dynamic compliance value was observed at the 30º position relative to the other angles, and the driving pressure was increased at head angles of 45º and 60º.
